# Supplementary material for: The interactome of intact mitochondria by cross-linking mass spectrometry provides evidence for coexisting respiratory supercomplexes
Source: Mol Cell Proteomics. 2017 Dec 8;17(2):216–32. doi: 10.1074/mcp.RA117.000470 (PMC5795388; doi:10.1074/mcp.RA117.000470)
Supplement: Supplemental Data [file supp_RA117.000470_133922_0_supp_23978_fzffwf.zip › spectra_annotation/mito_DR_spectra_annotation/81-2-83-1-107-1.pdf]

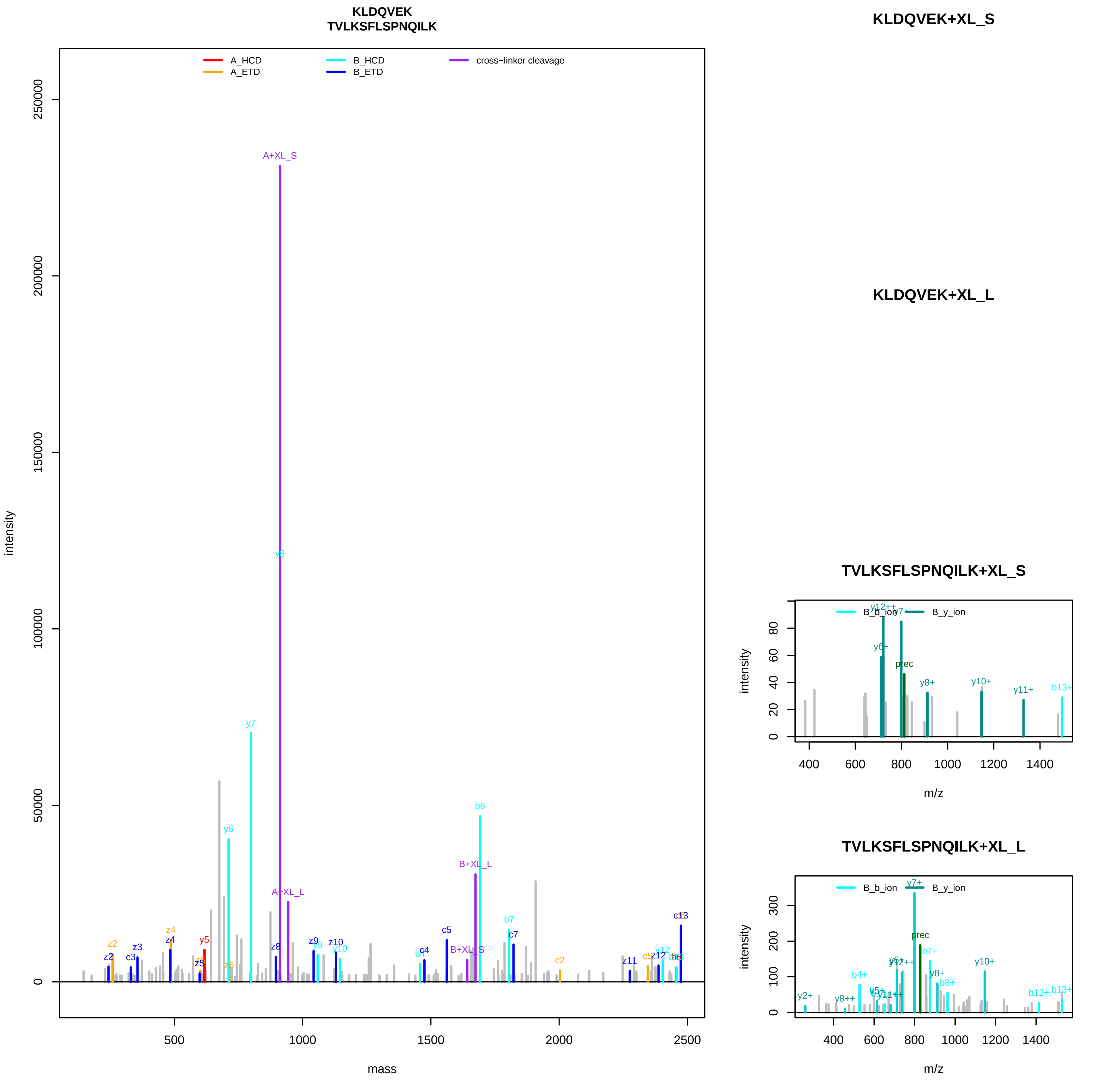

KLDQVEK
TVLKSFLSPNQILK
A_HCD
A_ETD
B_HCD
B_ETD
cross−linker cleavage
A+XL_S
A+XL_L
B+XL_S
B+XL_L
y5
y6
y7
y8
y9
y10
y12
b4
b6
b7
c2
c3
c4
c5
c7
c13
z2
z3
z4
z5
z6
z8
z9
z10
z11
z12
intensity
mass
0
50000
100000
150000
200000
250000
500
1000
1500
2000
2500
KLDQVEK+XL_S
KLDQVEK+XL_L
TVLKSFLSPNQILK+XL_S
B_b_ion
B_y_ion
y12++
y7+
y6+
prec
y8+
y10+
y11+
b13+
intensity
m/z
0
20
40
60
80
400
600
800
1000
1200
1400
TVLKSFLSPNQILK+XL_L
B_b_ion
B_y_ion
y7+
prec
b7+
y10+
y6+
y12++
b4+
y8+
b8+
y2+
y8++
y5+
y11++
b12+
b13+
intensity
m/z
0
100
200
300
400
600
800
1000
1200
1400
